# A bitter pill to swallow - Polypharmacy and psychotropic treatment in people with advanced dementia

**DOI:** 10.1186/s12877-022-02914-x

**Published:** 2022-03-16

**Authors:** Lina Riedl, Esther Kiesel, Julia Hartmann, Julia Fischer, Carola Roßmeier, Bernhard Haller, Victoria Kehl, Josef Priller, Monika Trojan, Janine Diehl-Schmid

**Affiliations:** 1 School of Medicine, Department of Psychiatry and Psychotherapy, Technical University of Munich, Ismaninger Str. 22, 81675 Munich, Germany; 2 School of Medicine, Hospital Pharmacy, Technical University of Munich, Munich, Germany; 3 School of Medicine, Institute of Medical Informatics, Statistics and Epidemiology, Technical University of Munich, Munich, Germany; 4 School of Medicine, Münchner Studienzentrum, Munich Germany, Technical University of Munich, Munich, Germany; 5 Neuropsychiatry, Charité – Universitätsmedizin Berlin and DZNE, Berlin, Germany; 6 University of Edinburgh and UK DRI, Edinburgh, UK

**Keywords:** YOD, LOD, Palliative care, Advanced dementia, Antipsychotics, Psychotropic drugs, Deprescribing, Polypharmacy

## Abstract

**Background:**

Polypharmacy is common in people with dementia. The use of psychotropic drugs (PDs) and other, potentially inappropriate medications is high. The aims of this cross-sectional study were 1) to investigate the use of drugs in people with advanced dementia (PWAD), living at home or in long term care (LTC); 2) to focus on PD use; and 3) to identify determinants of PD use.

**Methods:**

The study was performed in the context of EPYLOGE (IssuEs in Palliative care for people in advanced and terminal stages of YOD and LOD in Germany). 191 PWAD were included. All drugs that were administered at the date of the examination were recorded. Multiple logistic regression analysis identified determinants of PD use.

**Results:**

96% of PWAD received medication with a median number of four drugs. 49.7% received five or more drugs. According to the Beers Criteria 39% of PWAD ≥ 65 years received at least one potentially inappropriate medication. 79% of PWAD were treated with PDs. Older PWAD and PWAD living in LTC facilities received significantly more drugs than younger PWAD, and PWAD living at home, respectively. Dementia etiology was significantly associated with the use of antipsychotics, antidepressants and sedative substances. Place of living was associated with the use of pain medication. Behavioral disturbances were associated with the use of antipsychotics and sedative substances.

**Conclusions:**

To mitigate the dangers of polypharmacy and medication related harm, critical examination is required, whether a drug is indicated or not. Also, the deprescribing of drugs should be considered on a regular basis.

**Trial registration:**

Clinicaltrial.gov, NCT03364179. Registered 6 December 2017.

## Background

Although people with dementia are particularly vulnerable to medication related problems [1], a wealth of studies demonstrated that they suffer from polypharmacy and that the use of psychotropic drugs (PDs) and other, potentially inappropriate medications (PIMs) is high [2–6]. A standardized approach to prescribe and review medication in this complex and vulnerable population is lacking.

Because this population is often associated with advanced age, accompanying physical illnesses that require drug treatment are a factor that can complicate treatment. Specifically, multimorbidity can cause polypharmacy with the resulting problems of drug-drug interactions and side effects as well as adherence problems. The risk of inappropriate prescribing with consequential adverse drug events is increased [7–11].

These issues are combined with the problems of cognitive decline. Adhering to complex medication schemes and communicating adverse-drug reactions or difficulties with drug intake are associated with adverse health outcomes like emergency situations, hospitalisations and mortality [12].

The place of care appears to have an impact on medication as well. A French study highlighted the differences between older people with dementia (>75years old) living in LTC facilites compared to those cared for at home. Regardless of dementia severity, nursing home residents were significantly more likely to be prescribed anxiolytic, hypnotic, and antipsychotic medications [13].

Though dementia is typically a disease of older age, it does not exclusively affect older people. Symptom onset before the age of 65 years old is defined as young onset dementia (YOD).

Some types of dementia, e.g. Alzheimer’s disease, are treated with antidementia drugs. To control behavioral and psychological symptoms of dementia (BPSD), other psychotropic drugs are used. BPSD affect almost all people with dementia and result from disturbances in perception, thinking, feeling and behavior [14]. Especially antipsychotics (AP) are often used to treat BPSD, although they are associated with adverse health outcomes like cerebrovascular events, cognitive decline and higher mortality compared to people without dementia [14–19]. According to international guidelines, AP should be employed for the shortest duration possible after non-drug therapy, e.g. psycho-social intervention, have been deemed not effective [19–21]. Nevertheless, it has been shown that APs are often given as long-term drug therapy to people with dementia. The results of a nationwide, population-based French study imply that older people with dementia are chronically overexposed to PDs, particularly APs [22]. In fact, over half of the patients prescribed APs take them longer than 2 years [23].

Despite a large number of studies examining the influence of age at dementia onset and place of care in people with (typically old age) dementia throughout all disease stages, to our knowledge no current study has directly linked these influential factors specifically to advanced stages of dementia. Therefore, the aims of our study were to investigate the use of drugs, focusing on PDs, in people with young-onset dementia (YOD) and late-onset dementia (LOD) in advanced disease stages, living at home or in long term care (LTC) facilites. Further, we aimed to identify determinants of PD use in people with advanced dementia (PWAD).

## Methods

The prospective clinical study EPYLOGE (IssuEs in Palliative care for people in advanced and terminal stages of YOD and LOD in Germany) is the context in which this analysis was carried out. The study design of EPYLOGE has already been described [24], and results have recently been published [25]. Briefly, 191 individuals with YOD and LOD in advanced dementia stages were prospectively assessed at their place of living, either at home or in longterm care (LTC) facilities. The family caregiver was interviewed in a standardized manner. Medical and care files were used as additional sources of information. Prior to the recruitment of participants the study was approved by the Ethics Committee of the School of Medicine of the Technical University of Munich (18. Aug. 2017; No. 281/17 S). See [24] for detailed considerations about ethical issues, data management, quality assurance and data protection.

### Participants

People with advanced YOD or LOD, who were cared for at home or in LTC facilities, and the corresponding family caregiver were recruited. In order to achieve the goal of balanced group sizes (50% YOD and 50% LOD) stratification was carried out. Criteria that had to be fulfilled for inclusion were: 1) "Advanced dementia", defined as Clinical Dementia Rating (CDR, [20]) global score 2 or 3; 2) An adult family caregiver who was willing to participate; 3) Written informed consent from the family caregiver and the patient’s legal representative. The participants were recruited from previous patients who had been diagnosed with dementia since 2005 at the Center of Cognitive Disorders of the University Hospital of the Technical University of Munich.

### Assessments

A neurologist with psychiatric and palliative expertise (JH and CR) visited the PWAD at their place of living. The PWAD was thoroughly examined, the caregiver was interviewed in a standardized manner, and all available medical and care records were reviewed. Whenever care staff were involved, they were consulted as an additional source of information. Demographic data were gathered. The assessment included somatic, neurological, cognitive, and psychiatric symptoms. Documentation comprised the severity of dementia, quality of life, well-being/suffering, and drug therapy. The assessment tools are listed in Table 1 [25].

**Table 1:** Tests, interviews und questionnaires (alphabetic order) used for study purposes [25]

Name	Reference	Period of observation; type of survey	Interpretation
Barthel Index - activities of daily living	[26]	At the visit; Caregiver interview	10 subitems. Total score 0-100. The higher the better.
Clinical Dementia Rating Scale (CDR)	[27]	At the visit; Medical opinion based on the assessment of the PWAD and caregiver interview	Global score 0-3. Score 2: moderate dementia; Score 3: severe dementia
End-of-Life in Dementia Scale: Symptom Management (EOLD-SM)	[28]	Last 90 days; Caregiver interview	9 subitems. Total score 0-45. The higher the better symptom control.
Mini-Mental-State-Examination (MMSE)	[29]	At the visit; Cognitive test	Total score 0-30. The higher the less cognitive impairment.
Mini-Suffering-State-Examination (MSSE)	[30]	At the visit; Medical opinion based on the assessment of the PWAD and caregiver interview	10 subitems. Total score 0-10. The higher the more suffering.
Neuropsychiatric Inventory (NPI)	[31]	Last four weeks; Caregiver interview	12 subitems. Total score 0-144. The higher the more neuropsychiatric symptoms.
Pain Assessment in Dementia (PAINAD)	[32]	At the visit; 5-minute observation by study physician	5 subitems. Total score 0-10. The higher the more pain.
Quality of Life in Late Stage Dementia (QUALID)	[33]	Last week; Caregiver interview	11 subitems. Total score 11-55. The higher the lower quality of life.

Drug use was retrieved from medical files. All drugs that were prescriped at the date of the study assessment were recorded. Only regular prescriptions were recorded, since Pro Re Nata (PRN) prescriptions could not be reliably assessed, as they are hardly traceable or not reliably documented. Drugs were classified with the Anatomical Therapeutical Chemical classification [34]. For PWAD ≥65 years all drugs were evaluated according to the Beers Criteria [35]. PDs were grouped into pain medication (opioids N02A+ non-opioids N02B), antiepileptics (N03A), APs (N05A), anxiolytics (N05B), hypnotics (N05C), antidepressants (N06A) and antidementia drugs (N06D). For logistic regression PDs with sedative effects were combined into a group consisting of N05A (APs) + N05B (anxiolytic drugs) + N05C (hypnotic drugs).

### Statistical methods

The cross-sectional data evaluation approach includes descriptive analysis. The point prevalence of potentially inappropriate medication (PIM), according to the Beers Criteria [35], the number of drugs received and the percentage of polypharmacy (defined as more than four drugs) was determined for the day of the study assessment. For quantitative data, the mean and standard deviation (SD) as well as minimum and maximum values are reported. Continuous data of non normally distributed data were compared by the Mann-Whitney U test. Categorical variables were compared by CHI2 test. Multiple logistic regression analysis was performed in order to identify determinants of PDU. Odds ratios (OR) are reported along with 95% confidence intervals (CI). The following patient-related, independent variables were used: age, symptom onset, sex, etiology of dementia, place of living, Barthel-Index, Pain Assessment in Dementia (PAINAD), Quality of Life in Late Stage Dementia (QUALID), dementia severity as measured with Clinical Dementia Rating (CDR) sum of boxes, Neuropsychiatric Inventory (NPI), and End-of-Life in Dementia Scale: Symptom Management (EOLD-SM).

Based on the results of the multiple logistic regression analysis, an exploratory evaluation of the NPI was carried out subsequently to identify determinants of PDU on a symptom level. Imputation was used to replace missing values estimating them with a nearest neighbor method. Significance level was set at .05. Data were analyzed using Statistical Package for the Social Sciences (SPSS) 25 and Addinsoft (2021), XLSTAT statistical and data analysis solution, version 2020.5.1 .

## Results

### PWAD

191 PWAD were included in the study (49% with YOD, 51% with LOD). Severity of dementia was moderate (CDR global score = 2) in seven cases (4%) and severe (CDR global score = 3) in 184 cases (96%). Details of PWAD are provided in Table 2.

**Table 2:** Demographic data: age, symptom onset, sex, marital status, living situation, dementia etiology, dementia severity, functional and cognitive impairment, pain, behavioral symptoms and suffering Mean ± standard deviation (minimum-maximum)

PWAD age	74.1 ± 11.1 (40 - 101)	CDR sum score	17.3 ± 1.2 (11 – 18)
Symptom onset	YOD: 49% LOD: 51%	MMSE total score	1.5 ± 3.1 (0 - 14)
Sex	Female: 55% Male: 45%	Barthel-Index	26.9 ± 23.7 (0 - 85)
Marital status	Married/ in partnership: 68% Single: 32%	NPI total score	24.6 ± 17.0 (0 - 84)
Place of living	At home: 46% LTC: 54%	EOLD-SM total score	33.1 ± 7.6 (10 - 45)
Dementia etiology	AD: 65% FTLD: 24% Other: 11%	PAINAD total score	1.5 ± 1.8 (0 - 9)
If home care: family caregiver	Spouse/ partner: 62% Child: 29% Mother: 2%; Other: 7%	QUALID total score	21.6 ± 6.4 (11 - 44)
		MSSE total score	2.3 ± 1.6 (0 - 7)

*AD* Alzheimer‘s dementia, *CDR* clinical dementia rating scale, *EOLD-SM* end of life in dementia symptom-management scale, *FTLD* Frontotemporal lobar degeneration, *GDS* global deterioration scale, *HC* home care, *LTC* long term care, *LOD* late onset dementia, *MMSE* mini-mental-state-examination, *MSSE* mini-suffering-state-examination, *NPI* neuropsychiatric inventory, *PAINAD* pain assessment in dementia, *PWAD* person with advanced dementia, *QUALID* quality of life in late stage dementia, *YOD* young onset dementia

### Drug therapy

96% (*n*=183) of PWAD received at least one drug. 193 different drugs were administered. The twenty most frequent drugs were: 1. acetylsalicylic acid (19% of PWAD), 2. cholecalciferol (16%), 3. memantine (14%), 4. pantoprazole (14%), 5. bisoprolole (13%), 6. risperidone (13%), 7. levothyroxine-sodium (13%), 8. quetiapine (12%), 9. macrogol (12%), 10. torasemid (12%), 11. metamizole (11%), 12. ramipril (11%), 13. levetiracetam (10%), 14. mirtazapine (9%), 15. pipamperone (9%), 16. simvastatin (9%) 17. donepezil (9%), 18. sertraline (9%), 19. lactulose (7%), 20. lorazepam (7%).

### Potentially Inappropriate Medication (PIM)

According to the Beers Criteria [35], 39% of PWAD received at least one PIM. 19 % (n=37) of PWAD were treated with one PIM, 14% (n=26) with two, 6% (n=11) with three PIMs. Table 3 presents PIMs found in PWAD.

**Table 3:** Prevalence of PIMs according to Beers Criteria

**PIMs according to Beers Criteria**	
pantoprazole *n*=21; **quetiapine***n*=21; **risperidone**; *n*=20; **pipamperone**; *n*=15; **lorazepam** *n*=9; **melperone** *n*=9; **aripiprazole** *n*=4; insulin and analogues *n*=4; **zolpidem** *n*=3; omeprazole *n*=2; **paroxetine** *n*=2; **promethazine** *n*=2; **amisulpride** *n*=1; **clonazepam** *n*=1; **clozapine** =1; dimenhydrinate *n*=1; doxazosin *n*=1; **doxepin** *n*=1; **haloperidol** *n*=1; **hydroxyzine** *n*=1; **sulpiride** *n*=1; **tiapride** *n*=1	

Psychotropic drugs printed in bold

### Polypharmacy

The median number of drugs received by PWAD was four (min. 0; max. 15; 1^st^ quartile 2; 3^rd^ quartile 6). Eight PWAD received no regular medication, while one person regularly received 15 different drugs. 50% of PWAD (n=95) received 5 or more drugs. Details are shown in Table 4. Older PWAD (≥65 years old) received significantly more drugs than younger PWAD. PWAD in LTC facilities received significantly more drugs than PWAD who were cared for at home. Accordingly, polypharmacy was significantly more frequent in older PWAD and in PWAD who lived in an LTC.

**Table 4:** Number of drugs per PWAD and polypharmacy. Median (1^st^-3^rd^ quartile); Differences in regard to age group, living situation, sex, and symptom onset

		Median number of drugs received	*p*-value (MWU)	Proportion of polypharmacy (≥5 drugs)	*p*-value (CHI2)
**Total sample**	*N*=191	4 (2-6)		*n*=95; 50%	
**Age**					
<65 years	*n*=38; 20%	3 (2-5)	0.011*	*n*=13; 34%	0.032*
≥65 years	*n*=153; 80%	5 (3-7)		*n*=82; 54%	
**Living Situation**					
HC	*n*=88; 46%	4 (2-6)	0.001*	*n*=32; 36%	0.001*
LTC	*n*=103; 54%	5 (4-7)		*n*=63; 61%	
**Sex**					
female	*n*=106; 56%	5 (2-6)	0.480	*n*=57; 54%	0.213
male	*n*=85; 44%	4 (2-6)		*n*=38; 45%	
**Dementia onset**					
YOD	*n*=93; 49%	4 (2-6)	0.068	*n*=42; 45%	0.218
LOD	*n*=98; 51%	5 (3-7)		*n*=53; 54%	

*CHI2* chi square test, *HC* home care, *LTC* long term care, *LOD* late onset dementia, *MWU* Mann-Whitney-U test, *YOD* young onset dementia, * difference statistically significant

### Psychotropic drug treatment

79% of PWAD (n=150) received PDs. 26% of the participants (n=54) had one PD prescribed, 23% of PWAD (n=44) received two PDs and 28% of PWAD (n=52) got three or more. Details are shown in Table 5.

**Table 5:** Psychotropic drug treatment

Type of psychotropic drug (ATC-Level Code)	Number of drugs	PWAD (n)	% of PWAD
No psychotropic drug		41	21
ATC-Level 4 (N0XXX)	One psychotropic drug	54	26
	Two psychotropic drugs	44	23
	Three psychotropic drugs	27	14
	Four psychotropic drugs	17	9
	Five psychotropic drugs	7	4
	Six psychotropic drugs	1	1
Opioids (N02A)	One opioid drug	14	7
Non-opiods (N02B)	One non-opioid drug	23	12
Anti-epileptic drugs (N03A)	One anti-epileptic drug	38	20
	Two anti-epileptic drugs	1	1
Antipsychotic drugs (N05A)	One antipsychotic drug	55	29
	Two antipsychotic drugs	18	9
	Three antipsychotic drugs	1	1
Anxiolytic drugs (N05B)	One anxiolytic drug	15	8
Hypnotic drugs (N05C)	One hypnotic drug	12	6
Antidepressant drugs (N06A)	One antidepressant drug	62	32
	Two antidepressant drugs	3	2
Antidementia drugs (N06D)	One antidementia drug	44	23
	Two antidementia drugs	8	4
	Three antidementia drugs	1	1
Combinations of PDs	Combination of two types of PDs	49	26
	Combination of three types of PDs	31	16
	Combination of four types of PDs	8	4
	Combination of five types of PDs	1	1

*ATC* Anatomical Therapeutical Chemical classification, *PWAD* person with advanced dementia, *PD* psychotropic drug

A detailed overview of the treatment with psychotropic drugs is provided in Table 6.

**Table 6:** Psychotropic drug treatment

**Pain medication** (*n*=31; 16% of PWAD)	
metamizole (*n*=21; 11%); fentanyl (*n*=6; 3%); paracetamol (*n*=3; 2%); tilidine+naloxone (*n*=3; 2%); buprenorphine(*n*=2; 1%); morphine (*n*=2; 1%); cannabinoid (*n*=1; 0.5%); oxycodone (*n*=1; 0.5%);	
**Antiepileptics** (*n*= 39; 21% of PWAD)	
levetiracetam (*n*=19; 10%); valproic acid (*n*=7; 4%); pregabalin (*n*=6; 3%); lamotrigine (*n*=4; 2%); gabapentin (*n*=2; 1%); brivaracetam (*n*=1; 0.5%); carbamazepine (*n*=1; 0.5%); clonazepam(*n*=1; 0.5%); oxcarbazepine (*n*=1; 0.5%)	
**Antipsychotics** (*n*=74, 39% of PWAD)	
risperidone (*n*=24; 13%,); quetiapine (*n*=23; 12%); pipamperon(*n*=18; 9%); melperone(*n*=11; 6%); aripiprazole (*n*=5; 3%); clozapine (*n*=2; 1%); haloperidol(*n*=2; 1%); olanzapine (*n*=2; 1%); prothipendyl (*n*=2; 1%); zuclopenthixol (*n*=2; 1%); amisulpride (*n*=1; 0.5%); sulpiride (*n*=1; 0.5%); tiapride(*n*=1; 0.5%);	
**Anxiolytics** (*n*=15, 8% of PWAD)	
lorazepam (*n*=13; 7%); hydroxyzine (*n*=1; 0.5%); oxazepam (*n*=1; 0.5%);	
**Hypnotics** (*n*=12, 6%)	
zolpidem (*n*=4; 2%); melatonin (*n*=3; 2%); promethazine (*n*=2; 1%); zopiclone (*n*=2; 1%)	
**Antidepressants** (*n*=65, 34% of PWAD)	
mirtazapine (*n*=18; 9%,); sertraline (*n*=17; 9%); citalopram (*n*=11; 6%); escitalopram (*n*=7; 4%); duloxetine (*n*=5; 3%); venlafaxine (*n*=3; 2%); paroxetine(*n*=2; 1%); trazodone (*n*=2;1%); agomelatine (*n*=1; 0.5%); doxepin (*n*=1; 0.5%); tranylcypromine (*n*=1; 0.5%)	
**Antidementia drugs** (*n*=53, 28% of PWAD)	
memantine (*n*=27; 14%); donepezil (*n*=18; 9%); rivastigmine (*n*=11; 6%); gingko(*n*=3; 2%); galantamine (*n*=1; 0.5%)	

### Determinants of PDU

Results of the multivariate logistic regression analysis for determinants of PDU are presented in Table 7. For two out of 2156 data points missing data was imputed.

**Table 7:** Associations between patient-related parameters and psychotropic drug treatment If value 1 is not included in the 95% confidence interval, a significant association is indicated (*p* < 0.05); these results are printed in bold. 95% Confidence interval (lower boundary, upper boundary)

Parameters		Antipsychotics (N05A) OR (CI 95%)	Antidepressants (N06A) OR (CI 95%)	Sedatives (=antipsychotic + anxiolytic + hypnotic drugs) OR (CI 95%)	Pain medication (=N02A+N02B) OR (CI 95%)
Sex	male	0.97 (0.51-1.85)	0.86 (0.45-1.67)	0.94 (0.51-1.75)	0.49 (0.19-1.22)
	female	Reference			
Place of living	LTC	1.94 (0.96-3.93)	0.94 (0.47-1.90)	1.63 (0.83-3.18)	**3.32 (1.18-9.37)**
	HC	Reference			
Dementia onset	LOD	1.01 (0.37-2.82)	1.07 (0.37-3.08)	0.74 (0.28-1.98)	0.81 (0.21-3.14)
	YOD	Reference			
Dementia etiology	FTLD	**0.35 (0.15-0.83)**	1.15 (0.52-2.54)	**0.38 (0.17-0.86)**	0.98 (0.33-2.22)
	Others	1.01 (0.35-2.90)	**2.91 (1.04-8.16)**	1.23 (0.45-3.39)	0.34 (0.06-1.89)
	AD	Reference			
CDR (sum of boxes)		1.28 (0.87-1.88)	0.72 (0.51-1.03)	1.08 (0.76-1.53)	1.14 (0.60-2.18)
QUALID		1.03 (0.97-1.10)	1.03 (0.97-1.10)	1.04 (0.97-1.10)	1.02 (0.93-1.11)
PAINAD		1.04 (0.85-1.27)	0.96 (0.79-1.17)	0.99 (0.82-1.20)	1.25 (0.98-1.60)
Barthel-Index		1.01 (0.99-1.02)	1.00 (0.98-1.02)	1.00 (0.98-1.02)	0.99 (0.96-1.01)
NPI total		**1.04 (1.01-1.07)**	0.99 (0.96-1.01)	**1.04 (1.01-1.07)**	0.98 (0.95-1,02)
EOLD SM total		1.02 (0.97-1.07)	**0.94 (0.89-1.00**)	1.04 (0.99-1.09)	0.99 (0.92-1.06)
Age*		0.78 (0.49-1.25)	0.68 (0.41-1.11)	0.84 (0.53-1.32)	1.21 (0.64-2.29)

*AD* Alzheimer’s disease, *CI* Confidence Interval, *CDR* Clinical Dementia Rating scale, *EOLD-SM* End of life in dementia: Symptom-Management, *FTLD* frontotemporal lobar degeneration, *LOD* late onset dementia, *NPI* Neuropsychiatric Inventory, *OR* odds ratio, *PAINAD* Pain Assessment in Dementia, *QUALID* Quality of life in late stage dementia, *YOD* young onset dementia; *age was entered into the model with a multiplication factor of 0.1

First, multivariate analysis showed that BPSD as measured with the NPI as well as dementia etiology had a statistically significant association with the use of APs. A higher NPI total score (reflecting more BPSD) was associated with a higher prevalence of APs (OR: 1.04; 95% CI 1.01 to 1.07). FTLD, in contrast to AD or other dementia causes, was associated with a lower prevalence of AP.

Second, multivariate analysis showed that end-of-life symptoms as measured with the EOLD-SM as well as dementia etiology had a statistically significant association with the prevalence of antidepressants. A higher score in the EOLD-SM (reflecting better symptom control) was associated with a lower prevalence of antidepressants (OR: 0.94; 95%CI 0.89 to 1.00). Other dementia etiologies than AD and FTLD were associated with a higher prevalence of antidepressants.

Third, multivariate analysis showed that NPI as well as dementia etiology had a statistically significant association with the use of sedative substances. FTLD was associated with a lower prevalence of sedative substances, in contrast to AD or other dementia etiologies. A higher total score in the NPI (reflecting more BPSD) was associated with an increased use of sedative drug use (OR: 1.04; 95%CI 1.01 to 1.07).

Finally, multivariate analysis showed that the place of living had a statistically significant association with the use of pain medication (OR: 3.32; 95%CI 1.18 to 9.37). Living in a LTC facilities was associated with higher prevalence of pain medication.

Based on the results of the multiple linear regression analysis showing that the NPI was associated with the use of APs and sedative substances, an exploratory evaluation of the NPI was carried out subsequently in order to identify determinants of PDU on symptom level. The results are presented in Table 8 For 106 out of 2352 data points missing data was replaced.

**Table 8:** Associations between Neuropsychiatric Inventory subscores (frequency x severity) and psychotropic drug treatment If value 1 is not included in the 95% confidence interval, a significant association is indicated (*p* < 0.05); these results are printed in bold. 95% Confidence interval (lower boundary, upper boundary)

NPI subscores	Antipsychotics (N05A) OR (CI 95%)	Antidepressants (N06A) OR (CI 95%)	Sedatives (=antipsychotic + anxiolytic + hypnotic drugs) OR (CI 95%)	Pain medication (=N02A+N02B) OR (CI 95%)
Delusions	1.06 (0.91-1.23)	1.13 (0.99-1.30)	1.06 (0.91-1.23)	1.02 (0.84-1.24)
Hallucinations	1.00 (0.83-1.20)	0.93 (0.78-1.12)	0.96 (0.80-1.15)	0.76 (0.54-1.07)
Agitation/Aggression	0.96 (0.84-1.11)	0.99 (0.87-1.13)	0.95 (0.83-1.10)	1.12 (0.95-1.33)
Depression	0.92 (0.81-1.04)	1.04 (0.93-1.17)	0.91 (0.80-1.02)	1.08 (0.94-1.25)
Anxiety	**1.19 (1.04-1.36)**	1.03 (0.91-1.16)	**1.23 (1.06-1.43)**	1.05 (0.90-1.23)
Euphoria	0.93 (0.78-1.11)	0.99 (0.83-1.18)	0.90 (0.76-1.07)	1.10 80.88-1.38)
Apathy	1.01 (0.94-1.09)	1.02 (0.95-1.09)	1.00 (0.94-1.08)	1.04 (0.94-1.14)
Disinhibition	0.98 (0.86-1.12)	1.11 (0.99-1.25)	0.96 (0.85-1.09)	0.94 (0.79-1.13)
Irritability	1.13 (0.99-1.29)	0.93 (0.81-1.07)	1.10 (0.96-1.26)	0.95 (0.80-1.14)
Aberrant motor behaviour	1.03 (0.95-1.11)	0.97 (0.90-1.05)	1.03 (0.96-1.12)	0.93 (0.84-1.04
Nighttime behaviour	**1.10 (1.00-1.22)**	0.93 (0.84-1.03)	1.09 (0.99-1.20)	**0.84 (0.71-0.99)**
Eating disturbances	1.02 (0.94-1.12)	1.01 (0.93-1.10)	1.04 (0.96-1.14)	1.01 (0.91-1.13)

The multivariate analysis showed that an increase in anxiety (OR: 1.19; 95% CI 1.04 to 1.36)) and nighttime behaviour (OR: 1.10; 95% CI 1.00 to 1.22) was associated with higher prevalence of APs.

No statistically significant association between the use of antidepressants and the NPI was found.

An increase of anxiety was associated with a higher prevalence of sedatives (OR: 1.23; 95% CI 1.06 to 1.43). Further, an increase in nighttime behaviour was associated with a lower prevalence of pain medication (OR: 0.84; 95% CI 0.71 to 0.99).

## Discussion

### Drug use, potentially inappropriate medication and polypharmacy

In line with previous studies researched on this topic, we found the vast majority of PWAD were treated with more than one drug and polypharmacy was common with half of the PWAD receiving five or more drugs. Out of the 20 most frequently administered drugs, eight were PDs. Three drugs were preventive (acetylsalicylic acid, cholecalciferol, and simvastatine). According to Beers Criteria, 39% received PIM.

Unsurprisingly, older PWAD – who usually suffer from more physical illnesses [25] - received significantly more drugs than younger PWAD. Further, PWAD in LTC facilities received more drugs than those living at home. Similar results have been found in cohorts of older people (with and without dementia) [11]: Polypharmacy was common in older adults with the highest number of drugs taken by those residing in LTC facilities. A recent Italian study evaluated drug use in LTC facilities and reported the number of drugs prescribed were higher in residents without dementia than in those with dementia, the latter receiving 5.1 to 9.3 drugs per day [36].

The higher prevalence of drugs in LTC facilities as compared to home care, might be explained by the more comprehensive medical treatment in LTC facilities. In this type of care, a physician usually visits the PWAD at least once every three months, a higher frequency than those not in LTC facilities. Alternatively, PWAD may be admitted to LTC, when BPSD management at home becomes more difficult [37]. However, the study cannot answer if more intensive drug regimens in LTC actually are associated with better mental and physical health.

Lastly, there is a direct link between the number of drugs prescribed and the potential for dangerous drug-drug interactions, side effects and prescribing cascades. In Germany, specialized pharmacists infrequently are consulted for the treatment of people with dementia, to mitigate the chances of the unwanted consequences. We did not investigate, if prescribing physicians were aware of potential interactions or regularly checked for them in interaction databases .

### Psychotropic drug use

79% of PWAD in our study received PDs. This is similar to a study from the Netherlands [38] that reported a PD prevalence of 87% in people with YOD living in LTC facilities. Another Dutch study found a 52% prevalence of PDU in YOD living at home [39]. Van der Spek et al. reported a 60% prevalence rate in LTC residents with dementia [40].

The differences in terms of prevalence might be explained, by the different study populations (YOD vs LOD, home care vs LTC, people older than 70, etc.). Another important consideration is the definition of PD. This definition ranges from “nervous system drugs” (according to ATC classification) to indication reason. An example to illustrate this point is our decision to exclude antiepileptic drugs from the logistic regression model because the indication - sedation vs. anticonvulsion vs. both - could not be verified with certainty.

APs were the PDs that were most frequently prescribed in almost 40% of PWAD. As discussed above, the range of prevalences in APs found in other studies is comparable to differences in PDU. The atypical APs, risperidone and quetiapine, were the most frequent APs. Typical APs, like haloperidol, were rarely used, which indicates prescribing physicians were probably aware of their negative side effects, particularly parkinsonism. With 39% the point prevalence of APs in our study was relatively high. It is worthy to point out that even though these drugs have been shown to not only increase morbidity and mortality, but also have significant side effects like sedation and cognitive deterioration, are still prescribed so frequently. Although some PWAD can experience a relieve of burdening symptoms, such as anxiety or restlessness, when treated with APs, there is a risk in using them as AP treatment can reduce quality of life [25]. Alternatively, Mirtazapin and selective serotonin reuptake inhibitors (SSRI) were the most commonly prescribed antidepressants. Tricyclic antidepressants with their anticholinergic side effects, particularly on cognition, are often avoided, as recommended by several treatment guidelines [19, 21]. Although donepezile and rivastigmine are approved for the treatment of mild to moderate dementia in Germany, 15% of people with severe dementia received an cholinesterase inhibitor (CHE-I). The question of, if the prescribing physician hoped the CHE-I could have a positive effect on the patient even in advanced dementia or if the CHE-I were not deprescribed inadvertently remains open.

### Associations with psychotropic drug use

No associations were identified between the use of PD (antipsychotics, antidepressants, sedatives, and pain medication), and sex, symptom onset, severity of dementia, quality of life, pain, and impairment of basal activities of daily living.

A diagnosis of dementia due to FTLD was negatively associated with the use of APs and sedative substances. This is counterintuitive since BPSD are core features of behavioral variant frontotemporal dementia, the most common dementia in the FTLD spectrum. While BPSD could decrease in the advanced stages of frontotemporal dementia, BPSD in AD tends to increase in advanced dementia stages. Our analyses showed that “other” causes of dementia that mainly included vascular dementia and Lewy body dementia were positively associated with the use of antidepressants. It is challenging to compare our findings to other studies, mainly because the study design and the cohort differ considerably. In the aforementioned study of Koopmans et al. only people with YOD who were cared for at home were investigated [39]. PDU in total, defined as APs (N05A) + anxiolytics (N05B) + hypnotics (N05C) + antidepressants (N06A) were investigated. PDU was associated with age and depressive symptoms. Mulders et al. [38] who investigate YOD in LTC found that PDU in total (defined as in [39]) was associated with the male gender. This study also examined the PD subgroups and found an association between anxiolytics use and a measure of dementia severity. Specifically, anxiolytics were less frequently prescribed in less affected YOD. They also found a positive association between the administration of APs and non-Alzheimer's type dementia.

Bargagli et al. [41] addressed the question of predictors for AP treatment in dementia patients ≥65 years-old using a population-based approach. They found that people who were also treated with antidepressants or antidementia drugs were more likely to receive APs. PWD who received polypharmacy were treated with APs less likely.

Further, our data showed that being cared for in a LTC facility was positively associated with the use of analgesics suggesting that pain medications were better available, and the barrier to administer them were lower in the institutionalized setting than at home.

We found, that more BPSD, as measured with the NPI, were associated with a higher prevalence of APs. In particular, anxiety and nighttime behavior were associated with more AP treatment. This finding is difficult to interpret. It could mean BPSD are a consequence of AP treatment, or the AP treatment is not suitable or sufficient enough to alleviate the respective symptoms. Interestingly, inconspicuous nighttime behavior was associated with a decreased use of pain medication, which leads us to question if pain as a result from uncontrolled and negative nighttime behavior may have been overlooked, and therefore left untreated.

A limitation to our study is PRN drug use was not considered. PRN drugs could not be reliably assessed, as they were hardly traceable or not reliably documented in patient charts. The impression during most patient visits, however, was, that most LTC facilities were rather restrictive regarding PRN use, as were family caregivers of PWAD who lived at home. Another limitation to our study is the relatively low number of patients included. Many similar studies analyse large databases to come to their conclusion, however often lack a thorough characterization of the patients treated. With this in mind, the low sample size we used allowed us to carefully and meticulously analyse all parties involved.

## Conclusions

Taken together, our study demonstrated a high prevalence of polypharmacy in PWAD – particularly in older PWAD and in PWAD who were cared for in LTC facilities. Almost 80% of PWAD received PDs, 39% were treated with AP. AP use was associated with BPSD, particularly anxiety and nighttime behavior, but was not associated with quality of life, dementia severity, pain, impairment of activities of daily living, and burdensome symptoms at the end of life.

In order to avoid dangers of polypharmacy and medication related harm as best as possible, careful and critical examination is required. Futher, prescribers – particularly those prescribing AP and sedating drugs – have to acknowledge, that depescribing is an essential component of successful treatment. Last but not least, further studies are required to investigate the expectations of the effects and side effects somatic and PDs, in the particular group of people in late stage dementia.

## Abbreviations

AD: Alzheimer’s disease
AP: antipsychotic
ATC: Anatomical Therapeutical Chemical classification
BPSD: behavioral and psychological symptoms of dementia
CDR: Clinical dementia rating
EOLD-SM: end-of-life-in-dementia – symptom management
FTLD: Frontotemporal lobar degeneration
GDS: Global Deterioration Scale
HC: Home care
LOD: late-onset dementia
LTC: longterm care
MMSE: Mini-Mental-Status-Examination
MSSE: Mini-Suffering-State-Examination
NPI: neuropsychiatric inventory
OR: Odd’s ratio
PAINAD: Pain Assessment in Dementia
PD: psychotropic drug
PIM: potentially inappropriate medication
PWAD: people with advanced dementia
QUALID: Quality of Life in Late Stage Dementia
YOD: young onset dementia
